# The Vaccine Treatment of Pneumonia

**Published:** 1909-08-14

**Authors:** 


### Medicine.

### THE VACCINE TREATMENT OF PNEUMONIA.

A good deal of work is being done just now upon the vaccine treatment of pneumococcal affections, especially of pneumonia, and practitioners who are not yet familiar with the procedure are beginning to ask how it is done, and what are the results.

It is clear that it is necessary that the diagnosis should be correct; and although it may be relatively easy to determine that the patient is suffering from pneumonia it is much more difficult to be sure that that pneumonia is pneumococcal. It is true that the great majority of ordinary lobar pneumonias in adults are pneumococcal, but there are a sufficient number due to other organisms, such as the influenza bacillus, the pneumo-bacillus of Friedländer, the typhoid bacillus, and even others, to create some doubt in any particular case until cultural methods have proved that it really is the pneumococcus that is at work. Moreover, lobar pneumonia may be simulated by such other conditions as phthisis, subdiaphragmatic abscess, pleurisy with effusion, and so on.

### Points in Favour of Vaccine Treatment.

Granted, however, that the pneumonia is pneumococcal, or that the patient is suffering from the continued effects of a pneumococcal sequela of pneumonia, the reports that are being published upon the subject seem to indicate: first, that no harm results from the inoculations if ordinary skill and care are employed in giving them; and, secondly, that the course of the pneumonia is beneficially influenced by them. It would seem natural that the vaccine treatment should be begun as early as possible in each case, and this is strongly urged by those who advocate it at all; it would be unfair to employ every other method of treatment first, and then, simply because the patient was dying, and there seemed nothing else to be done, resort to vaccine treatment *in extremis*. Adopted early, and skilfully employed, it would seem that the vaccine treatment shortens the duration of the illness and increases the chances of recovery. It is important to remember, of course, that there is extreme variability in the virulence of the pneumococcus in different years, and that whereas in some epidemics of pneumonia all the patients recover whatever treatment is adopted, in others a large percentage of them die in spite of all that can be done. The pneumococcic vaccine treatment has yet to stand the test of time and more extended use; we are only giving the summary of the reports to hand so far.

### Dosage.

As regards the dose, a comparatively small one suits better than a larger one. A usual quantity of vaccine is that which contains the product of 50 million pneumococci; some observers prefer to start with 25 million and increase this in successive doses up to 150 million, or even more. In acute pneumonia there is little time for progressive increase of dose, however, so that a vaccine representing 50 million of the cocci is usual here; it is in the chronic empyemata and other pneumococcal sequelæ that larger and larger successive doses can better be employed.

### Index Determinations not Essential.

Another important question concerns the opsonic index. Is it necessary to determine the latter in order to decide when the dose of vaccine is to be repeated? The answer is "No." Determinations of the opsonic index may be helpful, but they are not essential. Observations of the temperature chart, of the clinical condition of the patient, and of the physical signs afford a sufficient guide in gauging the time when the dose should be repeated. It appears that intervals of four days are by no means too short when doses of 50 million are employed.

Next, as Dr. Butler Harris has recently insisted, chronic empyemata, and other infections of the lung or pleura by the pneumococcus which fail to resolve after an acute pneumonia, as well as pneumococcal lesions in other places, ought certainly to be treated with a pneumococcic vaccine. Slow to improve by themselves, or even stationary or retrograde, these cases seem to afford a very reasonable prospect of success when the patient's powers of anti-microbial reaction are given a fillip in the right direction by the use of the vaccine.

### The Origin of the Vaccine.

Finally it may be asked—and it must be admitted that the question is an important one—is it necessary that the patient's own pneumococci be used in preparing the vaccine? The answer is "No." It is true that in the treatment of furunculosis by anti-staphylococcic vaccines a great deal of stress has been laid upon the importance of making the latter from the patient's own organisms and not from stock cultures; such cultures admittedly give the best results in cases depending on streptococcic infection, and in such cases there can be no doubt that it is of the utmost importance to prepare a vaccine from the infecting micro-organism in the patient's tissues itself. Some authorities hold that it is equally important to do so in cases of pneumonia. The most recent opinions, however, seem to be that, provided the pneumococci have not been subcultured several times so as to have lost virulence, it matters little whether they were obtained directly from the patient or not. It is essential that the cultures used should possess a high degree of virulence, and to insure this it is as well to mix together several strains each as virulent as possible. Granted that they are virulent, however, it seems to matter little whence they are derived, and this is a point of considerable practical importance in that all the delay attendant upon cultivating the patient's own pneumococci can be avoided if the diagnosis is pretty obvious without it.

